# A Novel Human Biospecimen Repository for Clinical and Molecular Investigation of Thoracic Aortopathy

**DOI:** 10.3390/cardiogenetics11030017

**Published:** 2021-09-18

**Authors:** Courtney E. Vujakovich, Benjamin J. Landis

**Affiliations:** 1 Riley Hospital for Children, Department of Pediatrics, Division of Cardiology, Indiana University School of Medicine, Indianapolis, IN 46202, USA; 2 Department of Medical and Molecular Genetics, Center for Computational Biology and Bioinformatics, Indiana University School of Medicine, Indianapolis, IN 46202, USA

**Keywords:** thoracic aortic aneurysm, bicuspid aortic valve, aortopathy, Marfan syndrome, biobanking

## Abstract

Thoracic aortic aneurysm (TAA) is a heritable aortopathy with significant morbidity and mortality, affecting children and adults. Genetic causes, pathobiological mechanisms, and prognostic markers are incompletely understood. In 2015, the Collaborative Human Aortopathy Repository (CHAR) was created to address these fundamental gaps. Patients with thoracic aortopathy, associated genetic diagnoses, or aortic valve disease are eligible for prospective enrollment. Family members and controls are also enrolled. Detailed clinical and family data are collected, and blood and aortic tissue biospecimens are processed for broad usage. A total of 1047 participants were enrolled. The mean age in 834 affected participants was 47 ± 22 (range <1 to 88) years and 580 were male (70%). A total of 156 (19%) were under the age of 21 years. Connective tissue diagnoses such as Marfan syndrome were present in 123 (15%). Unaffected participants included relatives (*N* = 176) and healthy aorta tissue controls (*N* = 37). Aortic or aortic valve biospecimens were acquired from over 290 and 110 participants, respectively. RNA and protein were extracted from cultured aortic smooth muscle cells (SMCs) for 90 participants. Over 1000 aliquots of aortic SMCs were cryopreserved. The CHAR’s breadth, robust biospecimen processing, and phenotyping create a unique, multipronged resource to accelerate our understanding of human aortopathy.

## 1. Introduction

Thoracic aortic aneurysm (TAA) is an aortopathy that predisposes to aortic dissection, a life-threatening emergency. There is a strong heritable basis for TAA [1]. For example, Mendelian autosomal dominant connective tissue disorders including Marfan syndrome (MFS), Loeys–Dietz syndrome (LDS), and others have strong TAA associations. In addition, autosomal dominant causes of familial TAA have been identified in patients without syndromic characteristics. Turner syndrome (TS) is a genomic disorder that is commonly associated with TAA, and copy number variants (microdeletions or microduplications) account for a proportion of TAA [2,3]. In addition, bicuspid aortic valve (BAV) is the most common form of congenital heart disease and is frequently associated with TAA. Components of TAA pathogenesis have been elucidated from human genetics discoveries and mouse models [4]. Over time, molecular testing has begun facilitating organized approaches to prognosis and clinical decision making. Despite substantial progress, there remain key knowledge gaps in the areas of (1) genetic cause, (2) pathophysiology, and (3) clinical risk classification.

The Collaborative Human Aortopathy Repository (CHAR) study was created in late 2015 at Indiana University School of Medicine (IUSM). The study leverages the large, statewide clinical network of IUSM and existence of subspecialty programs dedicated to aortopathy care across all age ranges. The key objectives in the CHAR design were to (1) prospectively enroll patients with aortopathy for collection of human blood and aortic tissue biospecimens, (2) process and store biospecimens for multidimensional uses, and (3) acquire detailed clinical and family data to optimize the utility of biospecimens. By so doing, the CHAR is a unique, aortopathy-dedicated platform for investigations that seek to advance genetic understanding of TAA progression and cause, investigate molecular and cellular disease mechanisms, and develop clinical studies. In this report, we describe the CHAR study’s protocol and enrollment figures to date. We then discuss the basic and clinical questions that can be investigated using this platform, including current and future applications.

## 2. Methods

**Figure 1.**
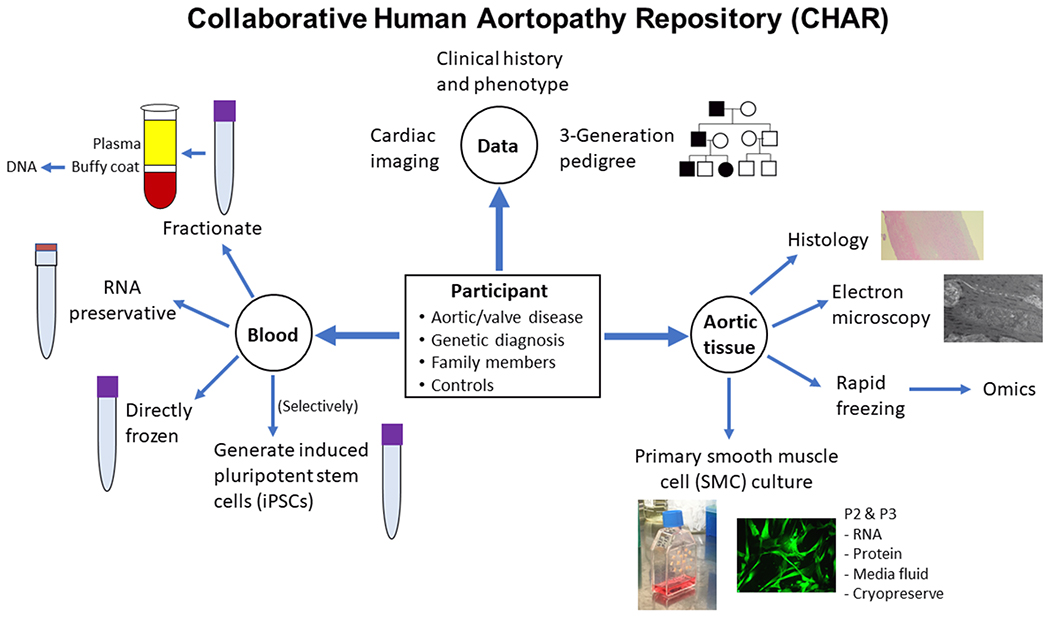
Synopsis of CHAR study’s eligibility, data collection, and processing of blood and aortic tissue biospecimens. P2: Passage 2; P3: Passage 3.

An overview of the CHAR study design is provided in Figure 1. The protocol was developed through discussions with a diverse array of clinical providers including cardiothoracic surgeons, medical geneticists, and cardiologists. Discussions were also held with clinical researchers with expertise in biobanking, technical directors of core laboratories, and other translational scientists. This study was approved by the Indiana University Institutional Review Board (Committee Reference Number: IRB00000219).

### 2.1. Eligibility and Enrollment Processes

In this prospective study, eligible patients are primarily identified via electronic medical record review at IUSM. Study personnel routinely review cardiothoracic surgery, genetics, and cardiology clinic rosters, as well as lists of admitted patients and operating room schedules, among the IUSM tertiary care facilities of Methodist Hospital, University Hospital, and Riley Hospital for Children. At Riley Hospital, a multidisciplinary aortopathy clinic was created and has expanded over the past 6 years to provide specialized, destination care to patients of all ages. The clinic’s providers are a highly integrated team of cardiologists, medical geneticists, and genetic counselors. A small number of participants (<5) have been enrolled outside of IUSM through public research avenues including clinicaltrials.gov and All IN for Health. The eligibility criteria are as follows:

- Diagnosis of aortic disease including TAA or dissection, aortic tortuosity, or aortic hypoplasia/stenosis.
- Diagnosis of a syndrome or genetic abnormality that poses risk for the development of aortopathy.
- Diagnosis of aortic valve disease.
- Family members of eligible subjects.
- Control subjects having aortic tissue removed during a surgical procedure, such as during heart transplantation.
- Organ donors who have authorized the use of their specimens for research.

In this study, TAA is defined by an absolute aortic diameter ≥ 3.7 cm or body surface area-adjusted *Z*-score value ≥ 2 by cardiac imaging that may include echocardiography, computed tomography (CT), magnetic resonance imaging (MRI), or conventional angiography. Syndromic diagnoses that meet eligibility criteria include, but are not limited to, MFS, LDS, TS, and vascular Ehlers–Danlos syndrome (vEDS). Aortic valve disease includes bicuspid, unicuspid, or tricuspid disease.

Eligible patients are approached in person or via telephone for opportunity to consent. Telephone consents follow a standardized script, to ensure that all study elements are discussed with the patient. Upon consent, study procedures are initiated. Sometimes patients have a medical emergency that requires immediate surgical intervention prior to the study staff being alerted of their medical condition. For these cases, the study staff provisionally acquire their biospecimens and basic clinical data. These patients are approached for consent and enrollment when they have recovered from their procedure and possess consent capacity. If the patient declines enrollment, their biospecimens and data are destroyed. If the patient dies prior to having the opportunity to obtain consent, the patient is permitted to remain enrolled. In the event that an enrolled participant dies, study staff may continue to collect data from the electronic medical record for research use.

### 2.2. Clinical and Family Data Collection

Table 1 summarizes the collected data categories and the main components of each. A customized database for clinical data was created in REDCap (Research Electronic Data Capture) for the purpose of this study [5,6]. It is an important priority of the study for data to be collected via a structured interview that occurs directly between clinical research staff and participants or their close family relatives (e.g., parents of children). This interview is accomplished in person or via phone. The database format is used to structure the interview. Data are entered directly into the REDCap database in real time using a tablet or computer. Any data that are uncertain or unavailable through the direct interviews are acquired in the electronic medical record, which is an integrated system across IUSM recruitment locations.

#### 2.2.1. Clinical Data

The cardiovascular-related data that are collected specifically are shown in Table 2. These include diagnosis and procedure history. The medication history data that are specified in the database to be recorded focus on cardiac medications. In non-cardiovascular data (Table 3), the components of a routine connective tissue evaluation comprise the majority of the characteristics that are collected. The non-cardiovascular characteristics of connective disorders MFS, LDS, and vEDS are included. This includes multiple systemic criteria for MFS in the revised Ghent nosology [7]. Characteristics that have been reported to be relatively frequent in LDS include bone fracture, osteoporosis, club foot, osteoarthritis, and cleft palate [8,9]. Characteristics associated with vEDS include bowel rupture, uterine rupture, recurrent hernia, and atrophic scarring [10]. Not all participants have undergone a genetics evaluation clinically. Therefore, the connective tissue characteristics that were thought to require a formal dysmorphology examination, such as facial features, pes planus, and hindfoot deformity, are not included in the interview.

#### 2.2.2. Family Data

A comprehensive family history is collected for each participant and entered as a three-generation pedigree. Similar to clinical data collection, the family history is acquired in person or over phone and utilizes a script. The script includes specific questions regarding cardiovascular history and risk, genetic testing and findings, and age/cause of death for deceased relatives. Non-cardiac family information, such as family members who have connective tissue characteristics, provided by the participant at the time of the interview is recorded. If a pedigree has been obtained clinically by a genetics provider, then that information is entered for this study. Any missing or new data since the time that the pedigree was last entered clinically are updated for this study.

### 2.3. Cardiac Imaging Data

#### 2.3.1. CT Scans

The results of all prior CT scans that include imaging of the aorta (chest, abdomen, pelvis, and occasionally neck) are collected. The data are collected directly from radiology reports and entered into a formatted spreadsheet. The procedures for collection of data from CT scans were recorded as an instructional video made available to research staff. For each scan, the body segments imaged and whether contrast was administered are recorded. Height and weight are recorded. The recorded vascular diameters are collected from all reported segments. A nomenclature for aortic segmental anatomy was adopted to encompass the range of reporting variation. Most collected scans were performed within the IU system, which minimizes such variation. Any diameter measured within a segment that has been previously repaired, replaced, or contains endovascular graft is denoted accordingly. The presence of arterial tortuosity and its specific location are recorded. Non-aortic arterial dilation/ectasia/enlargement, location, and diameters are collected. Other findings that are specifically recorded include elongation of transverse arch and venous ectasia. Additional cardiovascular abnormalities specified in reports, such as vascular/valvular calcification or atherosclerosis, are recorded. Aortic dissection is recorded and described as free text entry. Post-operative aortic changes such as pseudoaneurysms are also described. In addition to these cardiovascular data, incidental non-cardiovascular findings are also collected, such as abdominal organ cysts, hernia, and spine abnormalities. A customized downstream script was written in R (https://www.R-project.org; currently using version 4.0.4 access on 15 February 2021) to organize the spreadsheet data and automatically collate common anomalies that have been entered as free text.

#### 2.3.2. Echocardiograms

The diameters of the proximal aorta segments (annulus, root, sinotubular junction, ascending aorta) were directly measured by study investigators in a proportion of participants, including those who did not have CT scans completed clinically and young participants for longitudinal analysis.

### 2.4. Blood Sample Collection

Ambulatory peripheral blood samples are collected in clinical laboratories. In surgical cases where a blood sample was unable to be obtained prior to surgery, the sample is instead collected in the operating room prior to initiation of cardiopulmonary bypass. Blood is routinely collected into two purple-top EDTA tubes (approximately 6 mL per tube) and a 2.5 mL tube that contains RNA preservative (PAXGene; Hombrechtikon, Switzerland). An additional third purple-top tube is selectively acquired for generation of induced pluripotent stem cells from peripheral blood mononuclear cells [11]. Blood samples are immediately delivered to a core research laboratory (Clinical and Translational Support Laboratory) via the medical center’s interconnected tubing system. One purple-top EDTA tube is immediately placed into a −80 °C freezer. The second purple-top EDTA tube is centrifuged to fractionate the components, and the plasma (2 of 1 mL aliquots) and buffy coat are collected. According to the manufacturer’s instructions, the PAXGene tube is kept upright at room temperature for 2 to 72 h and then transferred to a −80 °C freezer. Samples are transferred in batches to the study laboratory for longer-term storage. Upon receipt, DNA is extracted from buffy coat samples and spectrophotometrically analyzed for quantity and quality.

#### Special Considerations

In children, a blood draw may collect no more than 3 mL per kg of body weight at one time. Multiple draws may be completed in order to collect the total desired amount of blood. Rarely, additional blood samples outside of the original 21 mL would need to be drawn from a participant of any age. Possible reasons for additional draws include sample loss, exhaustion of the original sample, or technical errors. If participants require additional draws, study staff will ask the participant to re-consent to the study. Participants who enroll into the study but are not willing to provide a blood sample are selectively offered the alternative to provide a saliva sample.

### 2.5. Tissue Sample Collection

When participants are scheduled for cardiac surgery that includes the removal of aortic and/or aortic valve tissue, tissue that will not be used clinically (i.e., sent to pathology) are collected for study in the operating room. Cardiothoracic surgeons and operating room staff were instructed on the protocol for processing explanted tissues. The study supplies for tissue processing are provided to operating room staff at the beginning of the case. An instruction sheet is always provided, which specifies in detail how an explanted tissue sample should be apportioned. A tissue sample information sheet is also provided, where collection data are recorded, including the time of explant and the time that the sample is processed.

Each aortic tissue sample is apportioned in four ways: formalin fixation, glutaraldehyde fixation, primary aortic cell culture, and rapid freezing. The collection is segmentally organized, such that, if a patient has more than one segment removed, then each segment is apportioned independently. The tissue samples are apportioned as quickly as possible.

#### 2.5.1. Formalin Fixation

A 1 × 1 cm piece is submerged in 10% formalin solution in the operating room. In the study laboratory, 24 to 72 h after collection, the formalin solution is changed to 30% ethanol for 1 h, followed by 50% ethanol for 1 h, and then the sample is stored in 70% ethanol at 4 °C until the time of paraffin embedding.

#### 2.5.2. Glutaraldehyde Fixation

A small 3 × 3 mm piece is placed into a microcentrifuge tube containing 4% glutaraldehyde with phosphate buffer in the operating room. Samples are kept at 4 °C until the time of embedding.

#### 2.5.3. Primary Aortic Cell Culture

A 2 × 2 cm piece is submerged in sterile aortic biopsy medium. The recipe for the medium was described by Kwartler et al. (https://bio-protocol.org/e2045; access on 1 June 2017). This tissue piece is promptly transported to the study laboratory for cell culture. Immediately upon receipt, an explant outgrowth method is utilized to culture primary aortic smooth muscle cells (SMCs). The adventitial and intimal layers of the aorta are dissected away from the medial layer. The medial layer tissue is cut into approximately 3 to 5 mm pieces and placed onto the surface of a cell culture flask (flask surface area of 25 cm^2^). The flask is then turned upright, and complete SMC growth medium is placed into the bottom of the flask, maintaining the fluid level below the tissue pieces. The complete SMC growth medium is composed of MCDB 131 (Gibco, Waltham, MA, USA) supplemented with glutamine, glucose, 5% fetal bovine serum (FBS), and the growth factors and antibiotics in the SmGM-2 Smooth Muscle SingleQuots Kit (Lonza, Basel, CH, Switzerland). The flask is placed upright into a sterile, humidified, 37 °C, 5% CO_2_ incubator. The flask remains upright for 2 h to allow for tissue attachment to the flask surface. After 2 h, the flask is repositioned flat so that the medium covers the tissue pieces. While tissue pieces are in the flask, the medium is changed every 3 to 4 days. When the outgrowing cells cover approximately 20% of the flask, which occurs approximately 3 weeks after the initial plating, the tissue pieces are removed and discarded, and the flask-adherent cells are trypsinized and subcultured according to the Lonza protocol.

Passaging, expansion, and routine subculturing steps are strictly protocolized to optimize consistency between primary SMC lines acquired from different participants. Passaging occurs every 7 days. At each passage, the cells are plated at a density of 5000 cells/cm^2^ into cell culture flasks (25 or 75 cm^2^ surface area) containing 1 mL per 5 cm^2^ of complete medium. Medium changes are performed the following day and then 3 days later, each time using 2 mL per 5 cm^2^ of fresh medium.

At the time of passages 2 and 3, cells are also plated into Nunc™ Cell-Culture-treated six-well plates (Thermo Scientific, Waltham, MA, USA). These cells are used for routine RNA and protein extraction. The cells are plated at concentration of 10,000 cells/cm^2^ in 5 mL/cm^2^ of complete medium per well. The medium is changed the following day with complete medium. Two days later, the complete medium is changed to low-serum medium (0.5% FBS) that does not have growth factor supplements. RNA and protein extractions are performed the following day. RNA is extracted using the RNeasy Mini kit (Qiagen, Germantown, MD, USA) from triplicate wells (yields three separate samples) and then aliquoted for storage at −80 °C. RNA samples are quantified by spectrophotometry with NanoDrop 2000 and a portion reverse-transcribed with the High-Capacity cDNA Reverse Transcription Kit (Applied Biosystems, Waltham, MA, USA). In parallel to RNA extractions, whole-cell protein lysates are extracted using ice-cold 1 × RIPA (abcam, Cambridge, MA, USA; ab156034) supplemented with 1% Halt™ Protease and Phosphatase Inhibitor Cocktail (Thermo Scientific). Adherent SMCs are washed twice with ice-cold PBS. After application of RIPA, the well surface is scraped and fluid is collected into cold microcentrifuge tubes by pooling the lysates of three wells of a six-well plate, before nutating at 4 °C for 60 min and centrifuging for 15 min at 12,000 rpm and 4 °C; then, the supernatant is collected. The supernatant sample is aliquoted and stored at −80 °C. During these extractions, the cell culture medium (i.e., extracellular fluid) is collected immediately prior to the first PBS wash. The collected media is centrifuged for 5 min at 300× *g* and 4 °C, and then the supernatant is stored at −80 °C.

At each routine passage, any excess cells not utilized for subculture or for RNA/protein sample extractions are cryopreserved in FBS and dimethyl sulfoxide. The cryovials are placed into a freezing-rate-controlled container (Mr. Frosty, Thermo Scientific) which is temporarily placed into a −80 °C freezer. Cryovials are transferred to liquid nitrogen tanks for long-term storage.

#### 2.5.4. Tissue Specimen Freezing

In the operating room, all of the tissue that remains after the apportioning described above is placed into a sterile Cryobag (OriGen, Austin, TX, USA). The Cryobags are immediately placed into a BioT™ ULT Transporter (Biocision, Larkspur, CA, USA) that has been prefilled with dry ice for rapid freezing. The biospecimens are then transferred to a −80 °C freezer. Frozen specimens are stored long-term in a customized drawer rack manufactured locally for Cryobag storage (Mid America Manufacturing Solutions, Mooresville, IN, USA).

#### 2.5.5. Aortic Valve Tissue

Aortic valve tissues undergo similar processing in the operating room, except that the cell culture is not routinely performed for aortic valves.

## 3. Results

A total of 1047 participants were enrolled into this study. These included 834 affected individuals and 213 who were not affected. The latter group consisted of relatives enrolled for family-based investigation (*N* = 176) and participants whose aortic tissue samples were used as healthy control tissues (*N* = 37). Table 4 summarizes the characteristics of affected participants. Patients with aortopathy were broadly eligible, but the predominant recruitment focus to date has been patients who have TAA or a genetic predisposition such as diagnosis of MFS. Consistent with epidemiological data showing that TAA is more common in males [12], there is a greater proportion of affected male participants than female. The racial and ethnic composition is generally consistent with the statewide demographics of Indiana. Approximately 50% of participants who are under the age of 21 years have a syndromic diagnosis. As expected, BAV is the most common congenital heart malformation in TAA cases. TAA is associated with less common congenital heart lesions such as tetralogy of Fallot and other conotruncal defects; however, these patients were not targeted for enrollment.

**Figure 2.**
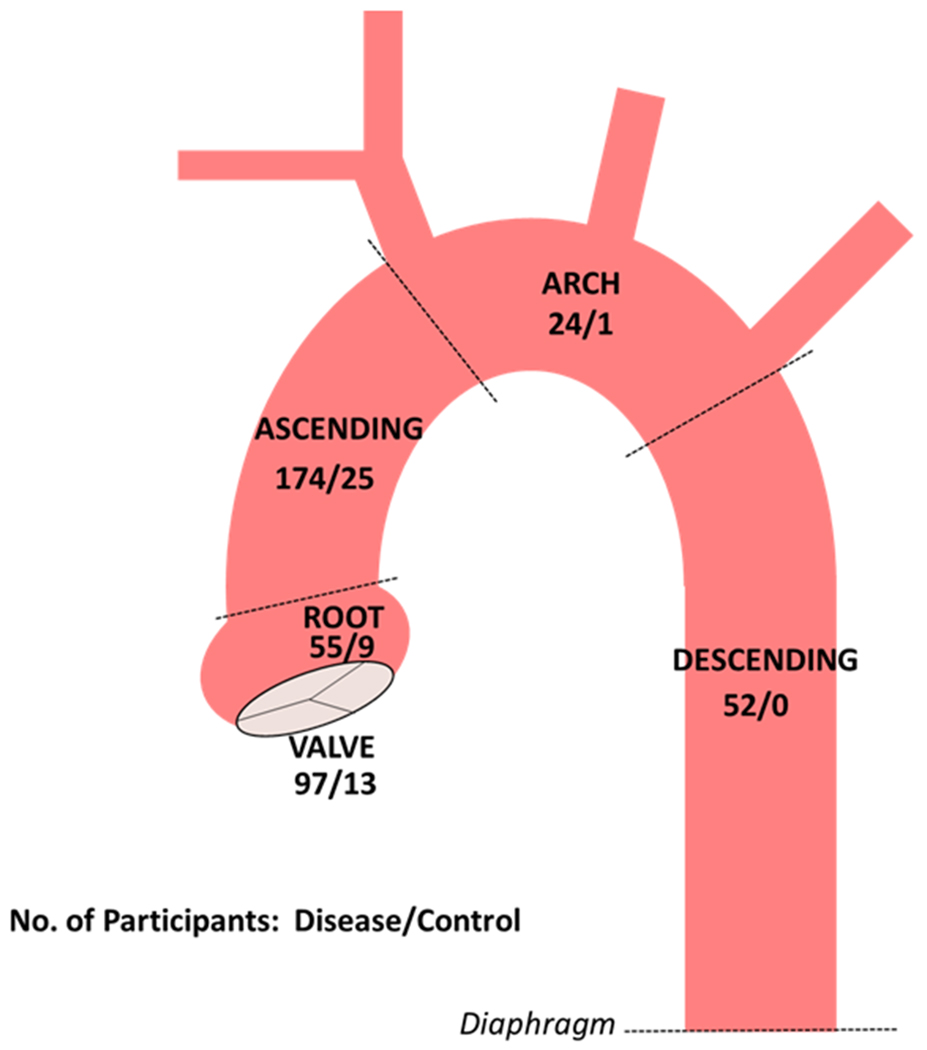
Numbers of aortic and aortic valve tissue samples collected to date in participants with aortopathy or aortic valve disease and normal controls. Dashed lines demarcate the boundaries of thoracic aortic segments.

As described in Methods, a direct interview with the participant or close relative (e.g., parent) is prioritized for all participants when available. To date, a direct interview has been accomplished for approximately 80% of participants. In total, 340 aortic tissue samples have been acquired from a total of 290 unique participants. The number of aortic tissue samples are shown across the different aortic segments in Figure 2. More than one segment has been collected from 48 participants. In most of such cases, the segments were acquired during the same operation. A total 110 aortic valve samples have been collected. RNA has been extracted from primary aortic SMCs following the routine protocol at passage 2 or 3 (usually both) for 96 participants. Protein has been extracted at passage 2 or 3 (also usually both) for 90 participants. The extracellular fluid has been collected for 68 participants at the time of cellular extractions. At least one aliquot of cultured aortic SMCs has been cryopreserved for 89 participants. In total, 1006 individual aliquots of cultured SMCs have been cryopreserved.

## 4. Discussion

There are myriad opportunities to investigate human aortopathy in the framework of the CHAR study. The successful development and implementation of the study protocol across adult and children’s hospitals required substantial coordination and collaboration in multiple clinical areas, including cardiology, surgery, and genetics. The repository has begun serving as a key resource for multiple lines of inquiry. The repository provides a powerful, human biology-centered platform to address important gaps in knowledge in aortopathy.

### 4.1. Incomplete Understanding of Genetic Causes of Aortopathy and Aortic Valve Disease

There are approximately 30 genes associated with TAA and dissection, and 11 of these genes were designated as definitive causes [13]. Understanding the biological roles for these genes and development of mouse models has increased the understanding of cellular and molecular mechanisms in heritable TAA [4]. Genotype–phenotype correlations have been developed over time, some of which have been adopted in clinical practice guidelines [14]. A genetic diagnosis is also important because it provides the opportunity to establish risk in family members through molecular screening. Despite significant advancements, currently known genetic causes account for fewer than 30% of familial cases. Thus, current clinical testing has limited yields, particularly in patients who do not have the signs for specific connective tissue disorders such as MFS. Inconclusive genetics evaluations commonly occur even in aortopathy patients who have connective tissue findings [15]. While there is clear evidence that congenital BAV is heritable, the known genetic causes account for a small fraction of cases [16]. Thus, there is a major need to identify novel causes of human TAA broadly, as well as associated aortic valve disease.

Many participants enrolled into the CHAR study do not have a genetic diagnosis established clinically. The detailed collection of family data using formal pedigrees and targeted family member enrollment will facilitate investigations for novel genes associated with TAA. Many CHAR participants provide both blood and tissue samples, creating an opportunity to directly investigate the mechanisms of suspected novel genetic causes that are identified. The comprehensive nature of the tissue and blood sample processing permits the effective investigation of functional effects in multiple ways. For example, cultured SMCs from many participants have already had RNA and protein extracted and SMCs have been cryopreserved, all in a protocolized manner. The prospective acquisition of clinical data and tissue samples will streamline phenotypic descriptions and functional investigations of genes and variants that are identified.

Clinically, variants of uncertain significance (VUSs) in genes that are known to be associated with TAA are commonly encountered [17]. We expect to identify VUSs through the course of genetic characterization of CHAR study participants. Here, too, the procurement of tissue samples matched to blood samples will facilitate direct investigation of the functional impact of VUSs. Functional evidence is part of the formal guidelines for interpretation of sequence variants from the American College of Medical Genetics and Genomics [18]. When VUSs are encountered clinically, functional studies are often unavailable or unfeasible. Instead, in silico methods are often utilized, but these rely on prediction algorithms that produce variable results, depend on a priori knowledge, and are not necessarily disease-specific. Where circumstances dictate, there is dedicated expertise in the multidisciplinary aortopathy clinic at Riley Hospital to report back and manage clinically actionable results, such as those encountered in prior aortopathy studies [19]. The integration between the CHAR investigators and personnel and the aortopathy clinic is also advantageous because many enrolled participants will have already had clinical testing, detailed phenotyping, and at-risk family members identified, as part of routine care. The application of genome sequencing in research and ultimately in clinical care will create a major need for resources to investigate the functional impact of potentially pathogenic genetic variants.

### 4.2. Undiscovered Mechanisms of Pathogenesis

Fundamental advances in the understanding of TAA pathogenesis have been achieved through the study of animal models. For example, in groundbreaking studies using a mouse model of MFS, Dietz et al. identified a novel mechanism of pathogenesis. Marked benefits of angiotensin II type 1 receptor blockade (ARB)- or transforming growth factor-β-neutralizing antibodies on aortopathy were demonstrated in genetically modified mice [20]. These results led to human clinical trials and the adoption of ARBs such as losartan as a medical therapy for patients with MFS [21]. While clinical efficacy has been demonstrated, the magnitude of the clinical effects of ARBs in humans may be less pronounced than in mice [22]. There are also instances of transgenic mouse models that do not completely recapitulate the human aortic phenotypes [23]. Interspecies variation in the pathobiology and genetic background, as well as different environmental conditions and exposures between laboratory animal experiments and human patients, may contribute to these incongruities. The mouse model work has been robust and highly informative, creating an opportunity to correlate the observations with human TAA pathogenesis. A more complete understanding of human pathogenesis will foster the identification of medical therapies and their indications, in order to ultimately cure TAA.

TAA is a genetically heterogeneous disorder. The degrees to which disease mechanisms may be distinct or converge between different etiologies is not well understood. Histologically, medial degeneration is a common finding [24], which supports a hypothesis that overlapping downstream mechanisms may exist. The scope of the CHAR study provides the capacity to compare clinical and genetic subtypes in novel ways. This opportunity is furnished by the tightly controlled and consistent manner in which the biospecimens are collected and processed. In addition, frozen tissue storage will facilitate omics-based analyses of split samples to integrate and correlate pathobiological data in multiple omics domains. Moreover, aortic biospecimens are collected from multiple aortic segments from the same individual participants when available. This will facilitate comparisons of pathological processes between segments, which has been proposed to be influenced by different developmental origins of SMC progenitors [25,26].

### 4.3. The Clinical Challenge of Risk Classification

Patients with TAA or genetic diagnosis often do not have cardiovascular-related symptoms until the sudden development of a life-threatening dissection. Medical therapy and other clinical interventions, including surgery, have benefits in TAA management but also pose associated risks and costs. Thus, TAA is a disease in which a precise stratification of an individual patient’s risk for progression and dissection would significantly improve clinical decision making. Currently, risk classification is imprecise in nature. While certain genetic diagnoses are associated with increased risk, such as MFS and LDS, there is still a large degree of interindividual variation in the severity of TAA within these groups, including between relatives [27]. The factors that influence penetrance and expressivity of genetic aortopathy are not well understood. Furthermore, it is estimated that only one-half of patients with a BAV develop TAA, also for reasons that are not understood. In our center, we have observed wide variability in disease progression between patients within different TAA subgroups starting at young ages [28]. This included the identification of some patients with BAV who progressed from normal aorta to significant TAA at early ages. While medical and surgical treatments are beneficial in TAA, more precise classification of risk will optimize outcomes and minimize harm.

The identification of genetic modifiers of aortopathy severity is a promising avenue toward optimizing risk stratification on a more individual basis [27,29]. Circulating biomarkers of disease progression may also help to understand and predict risk [30], but studies that include longitudinal sampling have been limited. We previously identified non-cardiovascular characteristics that were associated with the rate of aortic dilation in young TAA patients [15], which raised the possibility that systematic noncardiac phenotyping may have a role in the development of more precise prediction algorithms. Ultimately, the development of clinical prediction algorithms that incorporate genetic, molecular, and endophenotype attributes may lead to individualized clinical care and provide novel insight into pathobiological mechanisms. The design of the CHAR study is well situated to approach this fundamental gap.

### 4.4. Aortic SMCs: Cellular Disease Model and Substrate for Experimentation

Primary aortic SMCs are an established in vitro model for the study of aortopathy, as SMCs have a crucial role in the maintenance of extracellular matrix and vascular tone and SMC dysregulation is common in TAA. The large number of RNA and protein samples that have been extracted prospectively from SMCs at early passage, and the substantial cryopreservation effort (>1000 cell aliquots) will facilitate novel characterizations of SMC dysregulation. Additionally, experiments that include molecular or pharmacological manipulation of human primary SMCs provide results in the context of human biology.

### 4.5. Opportunities to Build upon the Foundation of CHAR

Participants in CHAR are well-phenotyped clinically. Investigational phenotyping techniques may be pursued to complement the clinical data. Examples include computational analysis of fluid dynamics and biomechanics for cardiovascular phenotyping and digital photography for craniofacial phenotyping. As the study increases in size and times-pan, development of electronic-based mechanisms for bidirectional communication with enrolled participants may improve the study’s monitoring of clinical and family status over time. Longitudinal blood sampling will enable biomarker studies for disease development or progression. Molecular characterization of the cohort with genetic testing performed on a research basis will optimize ongoing studies and prompt future investigations.

## 5. Conclusions

The CHAR study encompasses a large biobank specifically designed to investigate human aortopathy in a multidimensional manner. It provides a platform for novel investigation into genetic causes and modifiers and the mechanisms of pathogenesis and disease progression.

## Figures and Tables

**Table 1.** Categories of data that are routinely entered in the CHAR study.

Data Category	Components
Clinical data	Demographics Anthropometrics Medical history Surgical history Medication history Genetic evaluations
	Cardiac imaging performed

Family history	Three-generation pedigree (scripted)

Cardiac imaging	CT scans: Segmental aortic diameters; description of cardiovascular and non-cardiovascular findings Echocardiograms: Proximal aortic diameters; aortic valve function Cardiac MRI/MRA: Segmental aortic diameters

Biospecimen collection and processing	Blood Aortic/Valve Tissue

**Table 2.** Cardiovascular-related data collected through structured interviews and electronic medical record review.

Category	Diagnosis or Event (Y/N)	Details
Aortic	Thoracic aortic aneurysm	Location

	Thoracic aortic dissection	Date Stanford type (A vs. B)

	Thoracic aortic rupture	Date

	Abdominal aortic aneurysm	

	Abdominal aortic dissection	Date

	Abdominal aortic rupture	Date

Arterial	Brain aneurysm	Specify artery

	Aneurysm of other artery	Specify artery Diameter

	Dissection of other artery	Specify artery Date

	Rupture of other artery	Specify artery Date

Valvar	Aortic valve disease	BAV (Y/N) Aortic stenosis (Y/N) Aortic regurgitation (Y/N)

	Mitral valve prolapse	

Cardiovascular risk factors	Hypertension	Age of diagnosis Medical therapy (Y/N)

	High cholesterol	Age of diagnosis Medical therapy (Y/N)

	Diabetes	Type 1 vs. type 2 Age of diagnosis Medical therapy

Cardiovascular disease	Stroke	Ischemic versus hemorrhagic Date

	Coronary artery disease	Myocardial infarction (Y/N)

Venous thrombosis	Deep venous thrombosis	Specify vein Date

	Pulmonary embolism	Date

Heart rhythm	Arrhythmia	Type Date

	Syncope	Total number of lifetime episodes

	Cardiac arrest	Date

Congenital	Congenital heart disease (excluding BAV)	Specify

Other	Specify	

Procedures	Aortic replacement	Date Hospital Segments that were replaced Dissection present (Y/N)

	Aortic repair	Date Hospital Segments that were repaired Dissection present (Y/N)

	Endovascular repair	Date Hospital Segments that were repaired

	Aortic valve replacement	Date Hospital Simultaneous aortic replacement (Y/N)

	Mitral valve replacement	Date Hospital

	Mitral valve repair	Date Hospital

	Coronary artery bypass graft	Date Hospital

	Coronary artery stent or balloon angioplasty	Date Hospital

	Pacemaker or implantable cardioverter/defibrillator	Date Hospital

	Other	Specify

BAV: bicuspid aortic valve.

**Table 3.** Non-cardiovascular data collected through structured interviews and electronic medical record review.

Category	Diagnosis (Y/N)	Details
Skeletal	Pectus excavatum	Surgery required (Y/N)

	Pectus carinatum	Surgery required (Y/N)

	Joint hyperflexibility	

	Scoliosis	Age of diagnosis X-ray evaluation (Y/N) Surgery required (Y/N)

	Club foot	Right/left/bilateral Surgery required (Y/N)

	Joint pain	Specify joint

	Arthritis	Specify joint Surgery required (Y/N)

	Orthotics required before age 30	

Skeletal	Bone fracture	Specify bone Age when occurred

	Osteoporosis	

Ocular	Lens dislocation or subluxation	Right/left/bilateral Age of diagnosis Surgery required (Y/N)

	Vision correction (glasses or contact lens)	Age first prescribed Prescription strength Reason (e.g., near- or far-sighted)

	Retinal detachment	Right/left/bilateral Age of diagnosis

	Glaucoma	Right/left/bilateral Age of diagnosis

	Cataract	Right/left/bilateral Age of diagnosis

	Macular degeneration	Right/left/bilateral Age of diagnosis

Craniofacial	Orthodontia	

	Palate expander	

	Tooth extraction for dental crowding	

	Cleft palate	

Skin	Hyperextensible	

	Wide atrophic scar	

	Striae	Specify body location(s)

	Easy bruising	

Abdominal	Hernia	Location Surgery required (Y/N) Recurrence after surgery (Y/N)

	Eosinophilic esophagitis	Age of diagnosis

	Bowel rupture	Age of diagnosis

Microvascular	Frequent nose bleeds	

Immune	Kawasaki disease	Age of diagnosis Coronary artery dilation (Y/N)

	Vasculitis	Specify diagnosis Age of diagnosis

Development	Physical therapy in childhood	

	Learning disability	

	ADHD	

	Speech delay	

Neurologic	Seizure	Age Medical therapy (Y/N)

	Chronic headache	Migraine type (Y/N) Age when started

Pulmonary	Pneumothorax	Age Number of occurrences
		Spontaneous versus post-operative

	Emphysema	Age of diagnosis

	Asthma	Age of diagnosis Severity

Other medical diagnoses	Specify	

Other prior surgeries	Specify	

Obstetrical	Pregnancy	Number Number of births Number of spontaneous fetal losses

	Uterine rupture	

Lifestyle/social	Smoke cigarettes	Number of years Number of packs per day

	Other tobacco products	Specify type

	Regular heavy weightlifting for exercise	Number of years

	Regular heavy weightlifting for non-exercise purpose (e.g., occupational)	Number of years

The database includes the ability to enter additional details and comment where appropriate.

**Table 4.** Characteristics of the affected participants enrolled into the CHAR study.

Characteristic	Children and Adolescents (Age < 21 Years), *n* = 156	Adults, *n* = 678
Age at enrollment (years), mean ± SD	10 ± 6	56 ± 14

Sex, *n* (%)		
Male	99 (63)	481 (71)
Female	57 (37)	197 (29)

Race, *n* (%)		
White	145 (93)	630 (93)
Black or African American	6 (4)	39 (6)
Asian	4 (2.4)	7 (1)
Not available	1 (0.6)	2 (0.3)

Ethnicity, *n* (%)		
Non-Hispanic	152 (97)	669 (99)
Hispanic	4 (3)	7 (1)
Not available	0	2 (0.3)

Syndrome diagnosis, *n* (%)		
Marfan, *n* (%)	47 (30)	45 (7)
Loeys–Dietz, *n* (%)	12 (8)	7 (1)
Vascular-type Ehlers–Danlos, *n* (%)	7 (4.5)	5 (0.7)
Turner, *n* (%)	4 (3)	2 (0.3)

Thoracic aortic disease, *n* (%)		
Thoracic aortic aneurysm	110 (71)	577 (85)
Thoracic aortic dissection	0	151 (22)
Thoracic aortic rupture	0	5 (0.7)
Coarctation of the aorta	2 (1.3)	5 (0.7)

Bicuspid aortic valve, *n* (%)	53 (34)	170 (25)

Ventricular septal defect, *n* (%)	5 (3)	9 (1.3)

Atrial septal defect, *n* (%)	2 (1.3)	10 (1.5)

Percentages are of available data.
